# Characteristics of Gut Microbiome in the Murine Model of Pancreatic Cancer with Damp-Heat Syndrome

**DOI:** 10.3390/biomedicines12102360

**Published:** 2024-10-16

**Authors:** Yangbo Tong, Fang Han, Mengyao Liu, Tianyu Xu, Aiqin Zhang, Jiangjiang Qin, Yuhua Zhang, Xiang Qian

**Affiliations:** 1 The Second Clinical Medical College, Zhejiang Chinese Medical University, Hangzhou 310053, China; 201712201502011@zcmu.edu.cn (Y.T.); lmy627986862@163.com (M.L.); xtty31@163.com (T.X.); 2 Zhejiang Cancer Hospital, Hangzhou 310022, China; hanfang@zjcc.org.cn (F.H.); zhangaq@zjcc.org.cn (A.Z.); 3 Hangzhou Institute of Medicine, Chinese Academy of Sciences, Hangzhou 310018, China; jqin@ucas.ac.cn

**Keywords:** pancreatic cancer, damp-heat syndrome, murine model, gut microbiota

## Abstract

Purpose: Murine models of pancreatic cancer with damp-heat syndrome were established based on two methods to explore the differences in the composition of intestinal flora and to seek characteristic genera with potential for model evaluation. Methods: In our study, thirty-four C57BL/6J male mice were randomly divided into a control group (Con), a model group (Mod), a classic damp-heat syndrome group (CDHS), and a climate-chamber group (CC). CDHS and CC groups were fed with a high-fat diet and glucose water, while the CDHS group was given 2.4 g/kg alcohol by gavage for 10 days, and the CC group was placed in a climatic chamber with a set temperature of (32 ± 1) °C and humidity of (92 ± 2)% for 10 days. The Mod group, CDHS group, and CC group underwent tumor-building experiments on day 11. Tumorigenicity was then assessed twice a week. After 4 weeks, feces, colon tissue, and tumor tissue were taken from the mice and were tested, and the mice were euthanized afterwards. Results: Mice in the CDHS and CC groups showed symptoms similar to the clinical damp-heat syndrome observed in traditional Chinese medicine (TCM), and exhibited a worse general condition and more rapid tumor growth trend than those in the Mod group. The pathological examination indicated that inflammation was prevalent in the CDHS and CC groups. Both groups had a disrupted intestinal barrier and an overgrowth of pathogenic bacteria such as *c_Gammaproteobacteria*, *o_Enterobacteriales*, and *g_Bacteroides*. Their microbiota composition showed greater diversity. Conclusions: Intestinal flora may have a promising future in the discovery of indicators for evaluating a model of damp-heat syndrome in pancreatic cancer.

## 1. Introduction

Pancreatic cancer (PC) is one of the most aggressive malignancies [1], characterized by a poor prognosis [2] due to its insidious onset, difficulty in early diagnosis, and limited treatment options [3,4]. This malignancy originates from the exocrine and endocrine tissues of the pancreas, predominantly affecting ductal cells [5]. The molecular mechanisms driving PC involve genetic mutations [5], particularly in genes such as KRAS, TP53, and SMAD4 [6], and interactions with the tumor microenvironment (TME), including processes like epithelial-mesenchymal transition (EMT) [7]. These mutations and interactions disrupt normal cellular signaling, leading to uncontrolled cell proliferation, evasion of apoptosis, and the formation of a dense and immunosuppressed TME [8]. Furthermore, the pathophysiology of PC is exacerbated by extensive desmoplasia, which creates a fibrotic tissue barrier around the tumor, impeding drug delivery and immune system engagement [9]. This complex interaction between genetic mutations and microenvironmental factors contributes to the rapid progression of the disease and its resistance to treatment.

In traditional Chinese medicine (TCM), PC is often linked to damp-heat syndrome [10], a condition marked by the accumulation of dampness and heat within the body [11,12]. Damp-heat syndrome presents with symptoms such as emaciation, diarrhea, fatigue, jaundice, and a yellowish tongue, which are commonly observed in PC patients [13]. Recent studies have explored the scientific underpinnings of this syndrome, associating it with the inflammatory processes, metabolic dysregulation, and immune dysfunctions [14,15]. By aligning the pathophysiological mechanisms of cancer with TCM diagnostic principles, damp-heat syndrome provides a complementary framework for understanding the systemic imbalances involved in PC. This integration of TCM concepts with modern scientific research underscores the potential relevance of ancient practices in offering holistic approaches to cancer management.

The gut microbiome has become a crucial area of research [16,17], providing insights into the relationship between intestinal flora and overall health, including cancer development [18]. The composition of the intestinal microbiota influences immune function [19], metabolic processes [20], and inflammation [21], all of which are implicated in the pathogenesis of PC. One study [22] indicated that microbial dysbiosis in fecal samples from hepatocellular cancer patients was associated with unique microbial signatures, which could be used to distinguish cancer patients from healthy controls with good accuracy. This finding suggests that the gut microbiota offers potential as a noninvasive diagnostic indicator. With increasing evidence supporting the gut–cancer connection, leveraging the intestinal microbiome as a marker for early diagnosis, prognosis, and treatment response offers promising avenues for personalized cancer care [23].

## 2. Materials and Methods

### 2.1. Animal Preparation

A total of thirty-four specific pathogen-free (SPF) male C57BL/6J mice, aged 4 to 6 weeks and weighing between 18 g and 20 g, were obtained from Shanghai SLAC Laboratory Animal Co., Ltd. (Shanghai, China). They were housed under SPF barrier conditions at the Zhejiang Eyong Biomedical Research and Development laboratory animal facility (SYXK(Zhe)2021-0033, Zhejiang, China). The animals were kept under a 12 h light–dark cycle at a temperature of 22 ± 2 °C, with unrestricted access to food and water. This research was reviewed and given approval by The Ethics Committee for Animal Experimentation at Zhejiang Eyong Pharmaceutical Research and Development Center under authorization number ZJEF-20230720-03, dated 20 July 2023. All procedures adhered to relevant guidelines and animal usage regulations, following the ARRIVE guidelines (https://arriveguidelines.org, accessed on 14 July 2020). The detailed utilization of materials is provided in Table S1.

### 2.2. Induction of Damp-Heat Syndrome Models

The murine models were allocated into four distinct groups at random. Those in the Control group (Con, *n* = 6) and the Model group (Mod, *n* = 8) were maintained on a regular standard diet and had unrestricted access to tap water, with a 12 h light–dark cycle at a temperature of 22 ± 2 °C. The murine models in the classic damp-heat syndrome group (CDHS, *n* = 10) were given a 159.6 g/L glucose solution, a 45% high-fat diet, and 2.4 g/kg alcohol via gavage over a 10-day period [24]. Meanwhile, the mice in the climate-chamber group (CC, *n* = 10) were subjected to 159.6 g/L glucose and a 45% high-fat diet within a climate-controlled chamber under specific conditions: humidity at 92 ± 2%, temperature at 32 ± 1 °C, and a reversed 6 h dark to 18 h light cycle, all in an SPF setting, for 10 days [25].

### 2.3. PC Formation Experiment

**Figure 1:**
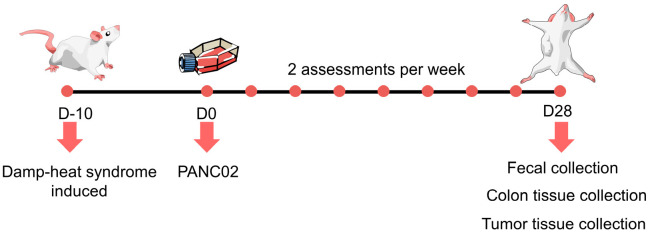
Pictorial representation of the study plan.

The Panc02 cell line, derived from mice, was sourced from iCell under authorization number iCell-m071 and grown in Dulbecco’s Modified Eagle Medium (DMEM) medium with 10% fetal bovine serum at a temperature of 37 °C. Once the cells reached near confluence, they were harvested. Panc02 cells (2 × 10^6^ cells in 100 μL saline) were subcutaneously injected into the mice belonging to the Model, CDHS, and CC groups [26]. The tumor’s long diameter (a, mm) and short diameter (b, mm) were recorded using a vernier caliper. Tumor volume was determined using the following equation: V = a × b^2^/2 [24]. Every four days, both the tumor volume and weight were assessed. At the end of the research, samples were gathered, images were captured, and the tumor size and mass were recorded and documented. After a period of four weeks, the mice were humanely sacrificed by performing cervical dislocation. The whole experimental procedure and its assessment are illustrated in Figure 1.

### 2.4. Histology

Colon and neoplastic tissues from mice were preserved in 4% formalin, encased in paraffin wax, and sliced into 5 μm thick sections. The tissue sections were stained with hematoxylin-eosin (HE) [27] and Masson’s trichrome [28]. ImageJ software (v1.8.0) was utilized to assess the extent of collagen fibrosis and inflammation in the colon. This procedure was carried out three times for consistency.

### 2.5. Immunohistochemical (IHC)

Tumor tissues from the murine specimens were fixed in 4% paraformaldehyde, encased in paraffin wax, and cut into sections 5 μm thick. After undergoing deparaffinization and rehydration, the sections were treated with citrate and blocked using 3% hydrogen peroxide (H_2_O_2_). Then, the slides were treated with 5% bovine serum albumin and subjected to primary antibodies at 4 °C over a period of 24 h. The primary antibodies employed included anti-α-SMA, anti-Ki67, and anti-FAP. Next, the sections underwent treatment with rabbit anti-goat IgG H&L and were stained using diaminobenzidine [29]. Hematoxylin was used for counterstaining. This process was repeated three times.

### 2.6. Immunofluorescent Staining

Tumor tissue slides were fixed in 4% paraformaldehyde for a quarter of an hour and then blocked with 5% bovine serum albumin at room temperature for an hour. The slides were then incubated overnight at 4 °C with primary antibodies, including anti-ZO-1 and anti-Occludin. After incubation, fluorescently labeled secondary antibodies were applied to the slides at 37 °C for 90 min. The samples were subsequently counterstained with DAPI for 6 min [29]. Fluorescence intensity was measured through ImageJ software. This procedure was performed in triplicate.

### 2.7. Processing of Samples and Bioinformatics Analysis of 16S rDNA

Freshly obtained fecal samples weighing 3–5 g were promptly stored at −80 °C for future analysis. Microbial DNA was isolated from these samples using the QIAamp Fast DNA Stool Mini Kit (QIAGEN, Hilden, Germany), adhering to the manufacturer’s instructions. Quality control of the extracted total DNA was carried out using agarose gel electrophoresis and the Thermo Nano-Drop 2000 UV spectrophotometer (Shanghai Yuanyao Biotechnology Co., Ltd., Shanghai, China). Sequencing of the V3–V4 variable regions from the eligible samples was conducted via the Illumina platform (Illumina, CA, USA). The raw sequences obtained post-sequencing were screened for quality, followed by separation into libraries and samples to eliminate barcode sequences. Operational Taxonomic Units (OTU) clustering was performed using the QIIME2 dada2 analysis pipeline. Alpha diversity analysis was employed to assess species richness and diversity within the samples. Principal coordinate analysis (PCoA), based on Bray–Curtis distance, was utilized to evaluate differences between groups. The Linear Discriminant Analysis Effect Size (LEfSe) method was applied to identify signature bacterial species [30,31].

### 2.8. Statistical Analysis

The study data were processed using SPSS version 20.0, with results expressed as means ± standard deviation (SD). Tukey’s test was used for statistical comparisons between groups. For comparisons across multiple groups, one-way ANOVA followed by Tukey’s test was applied. In cases where variances were unequal, the Kruskal–Wallis H test was utilized. A *p*-value of less than 0.05 was deemed to indicate statistical significance.

## 3. Results

### 3.1. General Status

**Figure 2:**
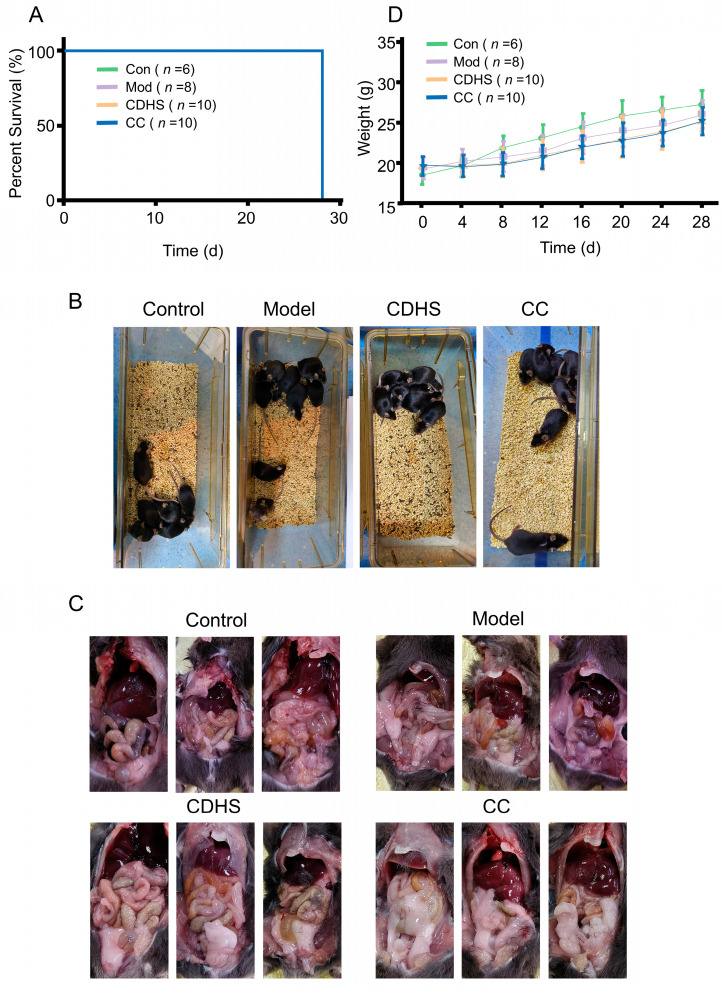
Evaluation of the damp-heat syndrome model in PC. (**A**) Percentage of survival over the course of 28 days. (**B**) Comparison of general health conditions among the various groups. (**C**) Accumulation of abdominal fat. (**D**) Weight variations in mice throughout the 28-day period.

There was no significant variation in overall survival rates across the different groups (*p* > 0.05, Figure 2A). Mice in the Control group demonstrated healthy general conditions, marked by glossy fur, active behavior, and an absence of unpleasant odor. In contrast, mice from the Model, CDHS, and CC groups showed signs of poor health, such as dull fur, lethargy, reduced mental alertness, constipation, and a noticeable odor (Figure 2B). Furthermore, abdominal fat accumulation was evident (Figure 2C), and both the CDHS and CC groups exhibited a plateau in body weight gain (*p* > 0.05, Figure 2D). The alterations in the CC group were more pronounced compared to the CDHS group.

### 3.2. Structural Alterations in the Colon’s Intestinal Barrier

**Figure 3:**
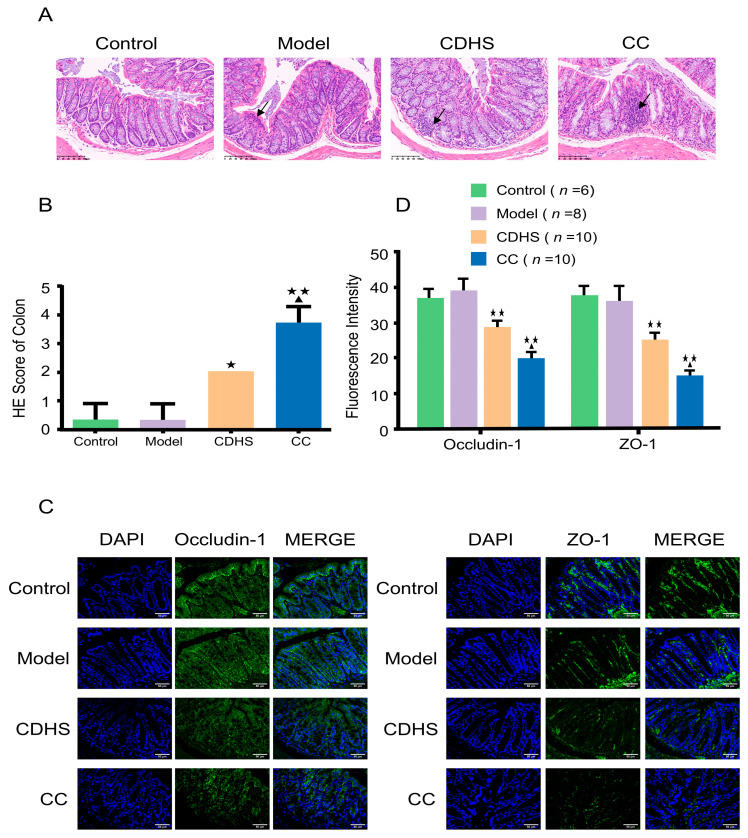
Alterations in intestinal barrier structure. (**A**) HE staining of colon tissues (magnification ×200, scale bar 100 μm). (**B**) HE scoring for colonic tissue. (**C**) Immunofluorescence staining of Occludin-1 and ZO-1 proteins (magnification ×200, scale bar 50 μm). (**D**) Fluorescence intensity analysis of Occludin-1 and ZO-1. Arrows point to regions of inflammatory cell infiltration. This procedure was conducted in triplicate. ^★^ *p* < 0.05, ^★★^ *p* < 0.01, CC or CDHS vs. Mod; ^▲^ *p* < 0.05, CC vs. CDHS.

In the Control group, HE staining of colonic tissues revealed a predominantly intact and normal structure. In contrast, the Model group exhibited infiltration of inflammatory cells, with the normal tissue architecture being only faintly discernible. Both the CDHS and CC groups displayed a disrupted mucosal structure along with significant infiltration of inflammatory cells. Moreover, the arrangement of glands in the colonic tissue from the CC group was found to be irregular (Figure 3A). Significant differences in HE scores were observed (*p* = 0.015, CC vs. Mod; *p* = 0.015, CC vs. CDHS, Figure 3B). Additionally, immunofluorescence analysis demonstrated a notable reduction in the fluorescence intensity of Occludin-1 and ZO-1 in the colonic tissues of mice from both the CDHS and CC groups (Figure 3C,D).

### 3.3. Tumorigenic Assessment

**Figure 4:**
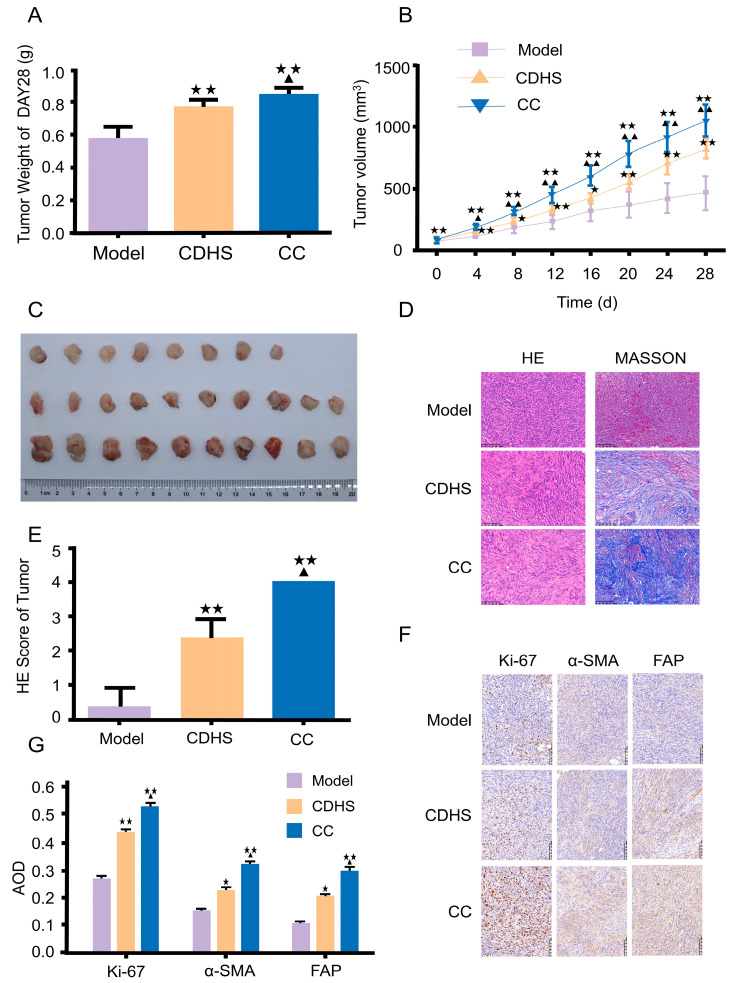
Tumorigenic evaluation. (**A**) Neoplastic weight on day 28. (**B**) Changes in tumor volume over time. (**C**) Neoplastic volume on day 28. (**D**) HE staining (magnification ×200, scale bar 100 μm) and MASSON staining (magnification ×200, scale bar 100 μm, Nikon Eclipse Ci-L). (**E**) HE score of tumor tissues. (**F**) Immunohistochemical analysis of neoplastic sections (magnification ×200, scale bar 100 μm). (**G**) Expression levels of Ki67, α-SMA, and FAP. The experiment was conducted in triplicate. ^★^ *p* < 0.05, ^★★^ *p* < 0.01, CC or CDHS vs. Mod; ^▲^ *p* < 0.05, ^▲▲^ *p* < 0.01, CC vs. CDHS.

To evaluate the impacts of damp-heat syndrome on PC, the primary focus was on measuring tumor size and weight. Tumor weight was found to be significantly higher in both the CDHS and CC groups compared to the Model group (*p* < 0.01, CC vs. Mod; *p* = 0.036, CC vs. CDHS, Figure 4A). Alterations in neoplastic volume mirrored the observed increases in neoplastic weight (*p* < 0.01, vs. CDHS, Figure 4B,C). The volume changes in single mice are presented in Tables S2–S9. HE and MASSON staining were conducted to assess the extent of collagen fiber proliferation (Figure 4D). The HE score indicated a notable disparity (*p* = 0.005, CC vs. Mod; *p* = 0.012, CC vs. CDHS, Figure 4E). Additionally, immunohistochemistry was performed to examine neoplastic proliferation activity. Neoplastic tissues from murine models in the CDHS and CC groups displayed expanded intercellular stroma and a higher presence of blue collagen fibers compared to the Model group (Figure 4F). The levels of Ki67, α-SMA, and FAP were markedly elevated in the CC group (*p* = 0.019, CC vs. Mod; *p* = 0.014, CC vs. CDHS, Figure 4G).

### 3.4. Characterization of Bacterial Flora

**Figure 5:**
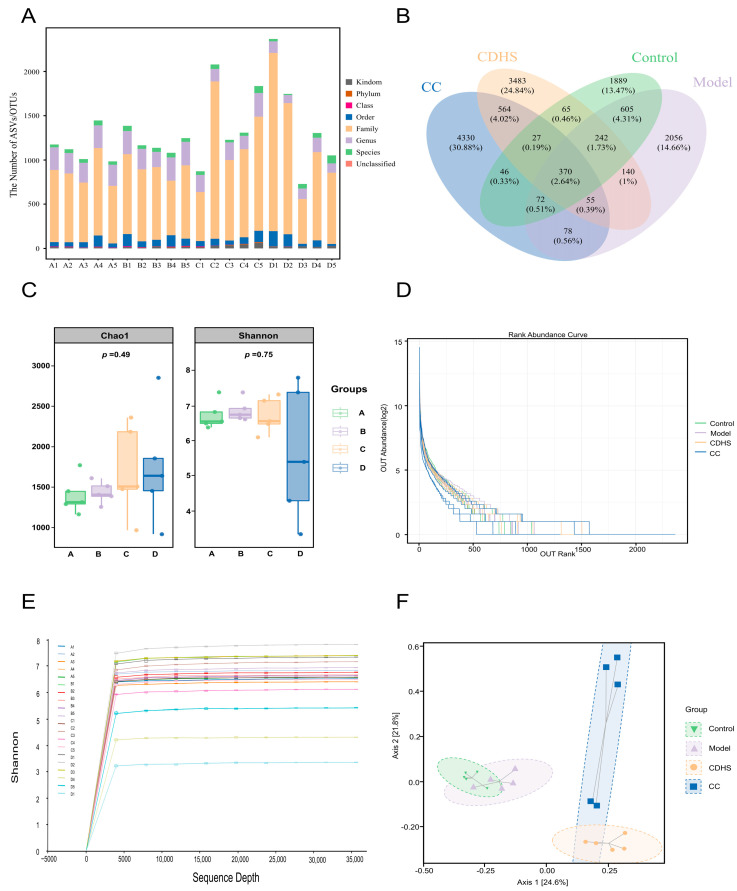
Results of species diversity across the four groups. (**A**) The count of OTUs in the four groups. (**B**) Venn diagram displaying the shared and unique OTUs among the groups. (**C**) Analysis of alpha diversity. (**D**) Rarefaction curve illustrating sequencing depth. (**E**) Abundance curve showing species richness. (**F**) PCoA plot representing beta diversity. Group A refers to the Control group, B to the Model group, C to the CDHS group, and D to the CC group.

This portion of the study included a total of 20 samples, each containing a diverse array of enriched OTUs (Figure 5A). The number of OTUs identified in each group were as follows: the Control group had 3316; the Model group had 3618; the CDHS group contained 4946; and the CC group had 5542 OTUs (Figure 5B). To assess alpha diversity, the Chao1 and Shannon indices were employed to reflect bacterial richness and diversity, respectively. Comparisons showed no significant differences in species diversity or abundance across the four groups (*p* > 0.05, Figure 5C). The rarefaction and abundance curves demonstrated that the sequencing depth was adequate and species annotation was comprehensive (Figure 5D,E). Using the Bray–Curtis distance algorithm, PCoA plots were created to visualize beta diversity, which revealed a clear trend of separation among the four sample groups (*p* = 0.001, Figure 5F).

**Figure 6:**
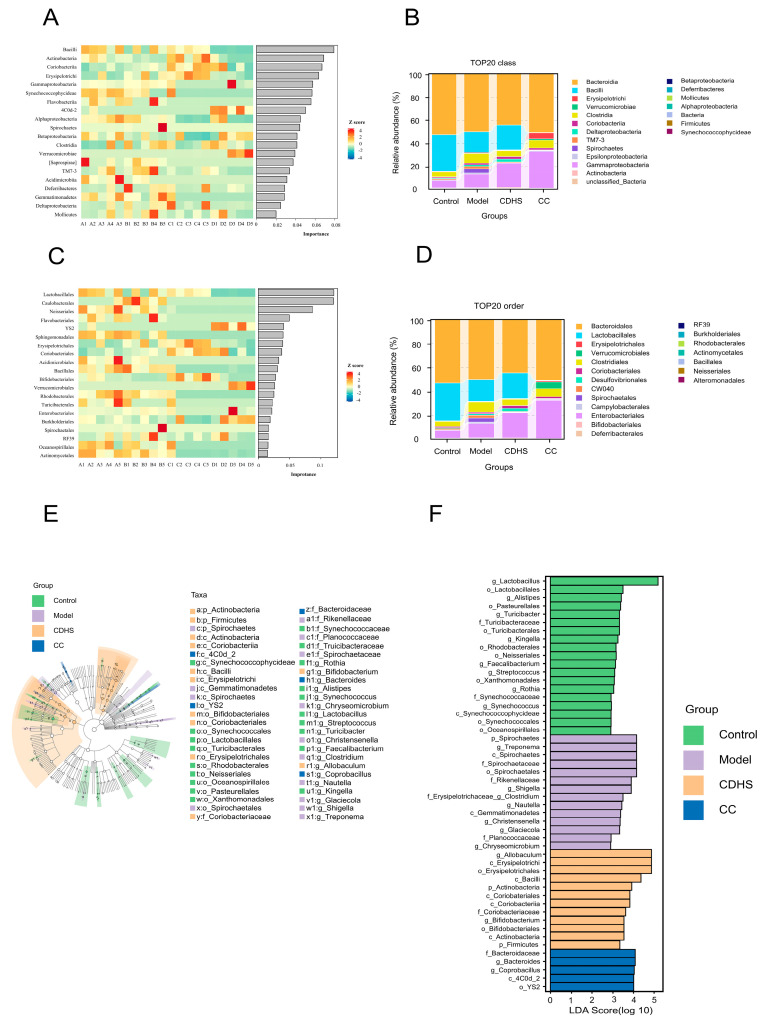
Bacterial flora characterization. (**A**) RFA at the class level. (**B**) Cumulative intragroup histogram at the class level. (**C**) RFA at the order level. (**D**) Cumulative intragroup histogram at the order level. (**E**) Taxonomic branch diagram generated by LEfSe analysis. (**F**) LDA score histogram.

Random forest analysis (RFA) was performed, along with a cumulative abundance assessment of the top 20 species within each group. At the class level, the CC group exhibited a higher relative abundance of *c_Gammaproteobacteria* compared to the other groups (Figure 6A,B). Likewise, at the order level, the CC group showed a significant increase in the relative abundance of *Enterobacteriales* (Figure 6C,D). The Linear Discriminant Analysis (LDA) effect size threshold was set at 4, with a p-value of 0.05 after adjusting for false discovery rate, to identify statistically significant microbial markers across the four groups. In the CC group, five microorganisms exhibited significant differences, ranked by LDA score from lowest to highest: *o_YS2*, *c_4C0d_2*, *g_Coprobacillus*, *g_Bacteroides*, and *f_Bacteroidaceae*. In the CDHS group, twelve distinct microorganisms were identified, ranked in ascending order by LDA score: *p_Firmicutes*, *c_Actinobacteria*, *o_Bifidobacteriales*, *g_Bifidobacterium*, *f_Coriobacteriaceae*, *c_Coriobacteriia*, *c_Coriobateriales*, *p_Actinobacteria*, *c_Bacilli*, *o_Erysipelotrichales*, *c_Erysipelotrichi*, and *g_Allobaculum*. In the Model group, fourteen microorganisms showed significant differences, listed by LDA score from lowest to highest: *g_Chryseomicrobium, f_Planococcaceae*, *g_Glaciecola*, *g_Christensenella*, *c_Gemmatimonadetes*, *g_Nautella*, *g_Clostridium*, *f_Erysipelotrichaceae*, *g_Shigella*, *f_Rikenellaceae*, *o_Spirochaetales, f_Spirochaetaceae*, *c_Spirochaetes*, *g_Treponema*, and *p_Spirochaetes*. Lastly, the Control group had nineteen microorganisms with significant differences, ranked from lowest to highest by LDA value: *o_Oceanospirillales, o_Synechococcales*, *c_Synechococcophycideae*, *g_Synechococcus*, *f_Synechococcaceae*, *g_Rothia*, *o_Xanthomonadales*, *g_Streptococcus*, *g_Faecalibacterium*, *o_Neisseriales*, *o_Rhodobacterales*, *g_Kingella*, *o_Turicibacterales*, *f_Turicibacteraceae*, *g_Turicibacter*, *o_Pasteurellales*, *g_Alistipes*, *o_Lactobacillales*, and *g_Lactobacillus*. Note that p, c, o, f, g, and s represent phylum, class, order, family, genus, and species, respectively (Figure 6E,F).

## 4. Discussion

In TCM, PC is categorized under conditions such as “accumulation”, “jaundice”, and “volvulus”. Dampness is further classified into internal and external dampness [32]. Based on this concept, we employed two distinct modeling methods. The internal dampness model (CDHS group) was induced by dietary factors combined with alcohol [24], while the external dampness model (CC group) was created using dietary factors combined with a hot and humid climate chamber [25,33]. There was no statistically significant difference between the two models in terms of survival rate. As for symptom presentation and tumorigenic assessment, mice in the CC group had better presentation than CDHS group. The analysis of intestinal flora revealed that the CC and CDHS groups harbored higher concentrations of pathogenic bacteria, including *c_Gammaproteobacteria*, *o_Enterobacteriales*, and *g_Bacteroides*, compared to the Model and Control groups. Conversely, the Control group exhibited a predominance of probiotic bacteria, notably *g_Lactobacillus*.

The potential of intestinal flora as a noninvasive, rapid diagnostic and prognostic tool has garnered significant attention in recent academic research. A notable prospective study from China revealed distinct disparities in the intestinal microbiota of patients with PC compared to healthy controls [16]. Further, another study demonstrated that combining fecal microbiome profiles, which are unique to PC, with biomarker CA199 enhances diagnostic precision, thus highlighting the feasibility of using microbial markers for non-invasive PC diagnostics [34]. Additionally, the role of gut microbiota in cancer pathogenesis has been explored, with particular focus on mechanisms such as leaky gut, bacterial ecological dysbiosis, and the influence of microbe-associated molecular patterns and bacterial metabolites in promoting hepatic inflammation, fibrosis, and genotoxicity that may drive cancer progression [35]. The potential for modifying gut microbiota through the use of probiotics, prebiotics, synbiotics, selective antibiotics, and herbal medicines to establish beneficial flora and prevent disease has also been documented [36]. Moreover, certain gut microorganisms have been identified to impact the efficacy of antitumor drugs. For instance, Geller et al. [37] employed 16S rDNA to elucidate the microbial composition associated with PC, identifying a predominance of microorganisms from *c_Gammaproteobacteria*, particularly *f_Enterobacteriaceae* and *f_Pseudomonadaceae*. These bacteria are capable of producing cytidine deaminase, which contributes to the degradation of and resistance to the chemotherapy agent gemcitabine. In a prognostic context, an analysis of a cohort of 1069 rectal and colon cancer cases [38] revealed that high levels of *Fusobacterium nucleatum* in tumor tissues were correlated with greater microsatellite instability and were prognostically linked to poorer outcomes, thus underscoring the prognostic relevance of gut microbes. These works not only advance our understanding of the microbiome’s diagnostic and therapeutic potential in cancer but also its prognostic implications.

Intestinal barriers consist of mechanical, biofilm, chemical, and immune components [39]. Intercellular tight junctions play a key role in maintaining the integrity of the mechanical barrier in the intestinal mucosa [40]. These tight junctions involve several proteins, including the zonula occludens (ZO) family, the claudin family, junctional adhesion molecules, and others [41]. Our findings indicated that the CC group exhibited the lowest expression of ZO-1 and Occludin-1 proteins. The downregulation of tight junction-related proteins compromises the physical barrier of the intestines, increasing intestinal wall permeability and facilitating the invasion of harmful bacteria and antigens. This exacerbates intestinal injury and inflammation, while impairing absorption, immunity, and endocrine functions. There is growing evidence supporting a biological interaction system between the gut microbiota and the pancreas, referred to as the microbiome–pancreas axis [42]. Studies have shown that patients with acute pancreatitis exhibit an increased proportion of *Bacteroidetes* and *Proteobacteria* in their gut microbiota [43]. In some cases, bacteria have been cultured from lymph nodes, plasma, and blood samples, most originating from the lower gastrointestinal tract, with *Escherichia coli* being the most common microorganism [44]. This supports the hypothesis that intestinal barrier disruption, or “leaky gut”, in an inflammatory environment can lead to bacterial translocation [45]. Our results further support this theory. Additionally, the Control group had a higher abundance of *g_Lactobacillus*, which improves intestinal inflammation, lowers cholesterol levels, enhances CD8 T cells infiltration, and inhibits tumor growth [46].

The transformation from inflammation to cancer is a prominent area of research, with pancreatitis being a well-established predisposing factor for PC [47]. Studies have demonstrated that the gut microbiota is associated with pancreas-specific diseases, such as pancreatitis and PC [42,48]. The PC microenvironment is characterized by hypoxia due to limited cellularity and the compressed stroma of bridging tissues [49], which promotes the growth of anaerobic bacteria [37]. Our findings are consistent with this observation, as *g_Bacteroides* and *g_Firmicutes* were significantly enriched in the mouse model of damp-heat syndrome. Intestinal inflammation was most pronounced in the CC group, followed by the CDHS group. In hypoxic conditions, mitochondria produce large amounts of reactive oxygen species (ROS), which can activate hypoxia-inducible factor-1α (HIF-1α) signaling. This activation enhances cancer cell migration and invasion through EMT [50] and also activates the proto-oncogene MYC via the ROS/S100A9/MYC signaling axis, promoting tumorigenesis. Additionally, dysbiosis in intestinal flora can lead to abnormal tryptophan metabolism, increasing 5-HT production. This activates the 5-HT/RhoA/NF-kB signaling axis, triggering downstream inflammatory responses that contribute to the early lesions of PC [51].

The TME in PC is characterized by a dense stroma, largely driven by cancer-associated fibroblasts (CAFs), which promote tumor growth, metastasis, and drug resistance [52]. The CC group exhibited the highest abundance of *c_Gammaproteobacteria* and *o_Enterobacteriales*, which are known to produce lipopolysaccharide (LPS), a component of their outer membrane [53]. Increased intestinal permeability allows LPS to translocate from the gut into the bloodstream, reaching pancreatic tissue. Once there, LPS binds to toll-like receptor 4 (TLR4) on CAFs and other stromal cells, activating the NF-κB signaling pathway [54]. This activation drives the expression of inflammatory ligands, growth factors, chemokines, extracellular vesicles, metabolite, EV-related molecules and so on, such as interleukin-6 (IL-6) [55], which further contribute to the EMT in cancer cells, enhancing tumor invasiveness, metastasis [56], therapy resistance [57], immune evasion [58], and relapse [59]. Additionally, activated CAFs secrete extracellular matrix proteins, including collagen and fibronectin, which form a hard matrix that disrupts the original vascular structure, promotes hypoxia, prevents drug penetration, inhibits immune cell infiltration, and thus promotes tumor growth [60].

To conclude, the CDHS and CC groups exhibited a significant enrichment in *c_Gammaproteobacteria* and *o_Enterobacteriales*. These microbial populations may facilitate the development of PC via multiple mechanisms, such as compromising the integrity of the intestinal barrier, enabling bacterial translocation, promoting inflammation, and activating CAFs.

In addition, this research presented several limitations: (1) It identified only an association between intestinal flora and PC in the context of damp-heat syndrome, without establishing a causal link; (2) the mechanisms involving gut microbiota were not thoroughly investigated; and (3) the small sample size in this study could introduce potential bias in the findings. Thus, future research should include a larger sample pool to enhance the reliability of the results.

## 5. Conclusions

In summary, we compared two damp heat murine models, evaluating their survival, symptoms, tumor growth, and the characteristics of their intestinal flora. We observed that the intestinal flora of mice subjected to dampness and heat included specific marker flora. Additionally, we explored the potential mechanisms through which these flora could promote tumor progression. Therefore, there exists a vast exploration space for incorporating intestinal flora as a model judging criteria, and it has strong exploration value as a non-invasive indicator of disease.

## Data Availability

The datasets generated and analyzed during the current study are available in the SRA repository with accession number: PRJNA1110560. Further information and requests for resources and reagents should be directed to the corresponding author.

## Supplementary Materials

The following supporting information can be downloaded at: https://www.mdpi.com/article/10.3390/biomedicines12102360/s1, Table S1. List of materials; Table S2. Tumor volume in single mice at Day0; Table S3. Tumor volume in single mice at Day4; Table S4. Tumor volume in single mice at Day8; Table S5. Tumor volume in single mice at Day12; Table S6. Tumor volume in single mice at Day16; Table S7. Tumor volume in single mice at Day20; Table S8. Tumor volume in single mice at Day24; Table S9. Tumor volume in single mice at Day28.
