# Supplementary material for: Characteristics of Gut Microbiome in the Murine Model of Pancreatic Cancer with Damp-Heat Syndrome
Source: Biomedicines. 2024 Oct 16;12(10):2360. doi: 10.3390/biomedicines12102360 (PMC11504882; doi:10.3390/biomedicines12102360)
Supplement: Supplementary file 1 [file biomedicines-12-02360-s001.zip › biomedicines-3252648-supplementary.pdf]

Table S1. List of materials.

Reagents or resources	Source	Identifier
Instruments		
Artificial climatic chamber	Ningbo Kesheng Experimental Instrument Co.,Ltd.	PRX-280B
IVC cages	Suzhou Suhang Technology Equipment Co. Ltd.	S9
Pulsating vacuum sterilizer	Zhangjiagang Valin Medical Equipment Co.,Ltd.	YXQ-MG-202
Water purification meter	Hangzhou Yajie Environmental Protection Equipment Co.,Ltd.	YJ-500L
Dehydrator	Shanghai Leica Instruments Co.,Ltd.	TP1020
Embedding machine	Tianjin Tianli Aviation Electromechanical Co.,Ltd.	BMJ-IB
Pathology Slicer	Shanghai Leica Instruments Co.,Ltd.	RM2235
Freezing table	Wuhan Junjie Electronics Co.,Ltd.	JB-L5
Tissue Spreader	Wuhan Junjie Electronics Co.,Ltd.	JK-5
Oven	Shanghai Sanfa Scientific Instrument Co.,Ltd.	DHG-9960A
Orthoptic microscope	Nikon (Japan)	Nikon Eclipse Ci-L
Imaging System	Nikon (Japan)	Nikon DS-Fi2
Panoramic Scanner	Ningbo Jiangfeng Biological	KF-PRO-120
Chemicals		
Ethanol	Guangdong Guanghua Technology Co.,Ltd.	20190318
Glucose	Guangxi Wuzhou Pharmaceutical Co.,Ltd.	NA
Neutral gum	Sinopharm Chemical Reagent Co.,Ltd.	10004160
Anhydrous ethanol	Sinopharm Chemical Reagent Co.,Ltd.	100092683
Xylene	Sinopharm Chemical Reagent Co.,Ltd.	10023418
Sodium chloride	Sinopharm Chemical Reagent Co.,Ltd.	10019318
Potassium chloride	Sinopharm Chemical Reagent Co.,Ltd.	10016318
Potassium dihydrogen phosphate	Sinopharm Chemical Reagent Co.,Ltd.	10017618
Disodium hydrogen phosphate (anhydrous)	Sinopharm Chemical Reagent Co.,Ltd.	2004061933
Hematoxylin	sigma	H3136

Eosin	sigma	E4009
Hematoxylin Stain	Runnerbio	Bry-0001-01
Differentiation solution	Runnerbio	Bry-0001-03
Rebluing solution	Runnerbio	Bry-0001-04
Glacial Acetic Acid	Sinopharm Chemical Reagent Co.,Ltd.	10000208
Acid compound red	Sinopharm Chemical Reagent Co.,Ltd.	71019360
Lichun Red	Sinopharm Chemical Reagent Co.,Ltd.	71033761
Hydrochloric acid	Sinopharm Chemical Reagent Co.,Ltd.	10011018
Aniline Blue	Sinopharm Chemical Reagent Co.,Ltd.	71003644
Phosphomolybdic acid	Beijing Wokai Biotechnology Co.,Ltd.	XW514297441
Neutral gum	Sinopharm Chemical Reagent Co.,Ltd.	10004160
Adhesive slides	Jiangsu Shitai Experimental Equipment Co.,Ltd.	188105
Coverslip (24*32mm)	Jiangsu Shitai Experimental Equipment Co.,Ltd.	10212432C
Blade	Japanese Feather	R35
3% H2O2	Sinopharm Chemical Reagent Co.,Ltd.	73113760
BSA	sigma	220107
Tris-base	sigma	WXBD176V
EDTA	sigma	P2050618
DAPI	abcam	ab104139
Antibodies		
Ki67 Antibody (1:100)	affinity	AF0198
alpha-SMA Antibody (1:200)	affinity	AF1032
FAP1 Antibody (1:100)	affinity	AF5344
Goat anti-rabbit IgG H&L (HRP) pre-adsorbed secondary antibody (1:5000)	abcam	ab97080
Occludin Antibody (1:100)	affinity	DF7504
ZO-1 Antibody (1:100)	affinity	AF5145

Goat Anti-Rabbit IgG H&L (1:500)	abcam	ab150079
Experimental Models		
C57BL/6J mice	Shanghai SLAC Laboratory Animal Co., Ltd.	SCXK (HU) 2022-0004
Cell lines		
Panc02	iCell Bioscience Inc Biotechnology Co., Ltd.	iCell-m071

Table S2. Tumor volume in single mice at Day0.

Groups	Tumor volume（mm^3）										
	1	2	3	4	5	6	7	8	9	10	P
Model	71.944	52.9515	82.8655	78.732	82.472	93.775	72.03	68.4285			
CDHS	91.854	89.7345	78.4195	95.506	85.2355	97.216	69.828	80.6285	75.118	108.9	0.144
CC	112.896	94.1015	93.312	71.5275	79.768	103.488	102.3435	119.422	95.2875	99.0945	0.214

Table S3. Tumor volume in single mice at Day4.

Groups	Tumor volume（mm^3）										
	1	2	3	4	5	6	7	8	9	10	P
Model	123.93	83.025	137.677	120.0945	122.786	141.312	107.3875	96.228			
CDHS	147.456	149.916	128.3355	160.55	157.604	159.3595	172.8265	146.202	139.606	187.272	0.001
CC	203.456	190.7825	166.8875	140.8995	170.582	174.3375	182.648	211.8645	175/071	183.2985	0.016

Table S4. Tumor volume in single mice at Day8.

Groups	Tumor volume（mm^3）										
	1	2	3	4	5	6	7	8	9	10	P
Model	206.388	121.2505	212.704	186.2935	204.723	267.696	166.698	137.924			
CDHS	194.0785	220.32	198.832	239.0625	241.056	255.881	255.528	241.968	237.65	279.738	0.032
CC	330.773	316.8	281.6275	255.881	282.123	280.136	306.5605	298.816	325.424	305.942	0.001

Table S5. Tumor volume in single mice at Day12.

Groups	Tumor volume（mm³）										
	1	2	3	4	5	6	7	8	9	10	P
Model	268.9965	151.424	263.7855	229.3655	261.5625	311.6475	217.328	169.3055			
CDHS	257.9115	299.568	284.0625	354.7835	333.8935	351.31	363.312	323.456	352	366.102	0.003
CC	479.2205	478.216	408.726	360.4055	410.504	393.7625	432.964	593.028	460.992	414.05	0.000

Table S6. Tumor volume in single mice at Day16.

Groups	Tumor volume（mm³）										
	1	2	3	4	5	6	7	8	9	10	P
Model	380.25	196.5645	354.294	341.0055	363.384	419.813	325.494	204.9125			
CDHS	343.1875	384.592	384.552	439.6155	408.2125	450.636	455.7625	420.229	436.364	481.562	0.019
CC	646.866	676	557.568	494.606	556.9725	534.578	588.7995	776.223	629.865	548.856	0.000

Table S7. Tumor volume in single mice at Day20.

Groups	Tumor volume（mm³）										
	1	2	3	4	5	6	7	8	9	10	P
Model	451.2295	217.5625	414.176	397.3725	395.686	505.4	358.4	222.2055			
CDHS	454.0725	501.642	534.578	585.844	509.2815	604.713	582.624	542.5885	566.5095	654.368	0.001
CC	812.879	868.292	711.1125	646.07	760.266	700.194	757.7415	1012.112	814.4955	712.86	0.000

Table S8. Tumor volume in single mice at Day24.

Groups	Tumor volume（mm³）										
	1	2	3	4	5	6	7	8	9	10	P
Model	530.464	238.144	475.26	447.174	469.752	552.23	419.832	240.4305			
CDHS	588.7995	637.5625	691.866	752.812	641.9655	751.581	799.254	654.3125	681.1695	821.632	0.000
CC	944.906	998.3505	837.828	778.909	900.4125	813.8485	1030.376	1156.5495	893.101	826.5625	0.000

Table S9. Tumor volume in single mice at Day28.

Groups	Tumor volume（mm^3）										
	1	2	3	4	5	6	7	8	9	10	P
Model	571.05	257.985	555.0375	505.4	508.07	613.836	457.9245	259.6115			
CDHS	740.096	778.2055	834.9	922.716	726.7165	867.912	912.022	729.904	804.825	944.541	0.000
CC	1086.072	1145.512	992.4525	867.912	1007.128	955.206	1180.062	1310.788	991.752	948.787	0.000
